# Diagnostic performance of Gd-EOB-DTPA-enhanced MRI for evaluation of liver dysfunction: a multivariable analysis of 3T MRI sequences

**DOI:** 10.18632/oncotarget.26368

**Published:** 2018-11-20

**Authors:** Niklas Verloh, Kirsten Utpatel, Florian Zeman, Claudia Fellner, Hans J. Schlitt, Martina Müller, Christian Stroszczynski, Matthias Evert, Philipp Wiggermann, Michael Haimerl

**Affiliations:** ^1^ Department of Radiology, University Hospital Regensburg, Regensburg, Germany; ^2^ Department of Pathology, University Regensburg, Regensburg, Germany; ^3^ Center for Clinical Trials, University Hospital Regensburg, Regensburg, Germany; ^4^ Department of Surgery, University Hospital Regensburg, Regensburg, Germany; ^5^ Department of Internal Medicine I, Gastroenterology, Endocrinology, Rheumatology, and Infectious Diseases, Regensburg University Hospital, Regensburg, Germany; ^6^ Department of Radiology and Nuclear Medicine, Hospital Braunschweig, Braunschweig, Germany

**Keywords:** magnetic resonance imaging, liver, abdomen, MELD score, multiparametric examination

## Abstract

**Objective:**

The aim of this study was to evaluate the diagnostic performance of a multiparametric gadolinium ethoxybenzyl-diethylenetriaminepentaacetic acid (Gd-EOB-DTPA)-enhanced MRI examination for the estimation of liver dysfunction classified by the Model for End-Stage Liver Disease (MELD) score.

**Results:**

Liver dysfunction can be assessed by different methods. In a logistic regression analysis, T1- and T2-weighted images were affected by impaired liver function. In the assessment of liver dysfunction, the reduction rate in T1 mapping sequences showed a significant correlation in simple and multiple logistic regression.

**Conclusion:**

Changes in Gd-EOB-DTPA-enhanced MRI between plain images and images obtained during the hepatobiliary phase allowed good prediction of liver dysfunction, especially when using T1 mapping sequences.

**Materials and Methods:**

A total of 199 patients underwent contrast-enhanced MRI with a hepatocyte-specific contrast agent at 3T. In the multivariable analysis, the full range of available MRI sequences was used to estimate the liver dysfunction of patients with various MELD scores.

## INTRODUCTION

The assessment of liver function is essential for determining the prognosis and clinical management of patients with chronic liver disease and for patients undergoing liver surgery [1, 2].

Several tests have been proposed or are used in daily clinical practice to assess liver function, ranging from tests based on laboratory values to metabolic tests. A widely used assessment is the Model for End-Stage Liver Disease (MELD) score. The MELD score combines several biochemical values (serum bilirubin, serum creatinine, and the international normalized ratio for prothrombin time) to determine liver function, serving as an indicator for patient treatment [3]. The most common metabolic test is the indocyanine green (ICG) clearance test, which uses an optical measurement technique to determine the blood clearance rate of intravenously injected ICG [4, 5]. New non-invasive technics are rising to analyze liver fibrosis, for example, Afdhal *et al.* showed that FibroScan (vibration-controlled transient elastography) provides an accurate assessment of liver fibrosis in patients with hepatitis B or C in comparison to histology [6]. These tests are suitable for measurement of global liver function; however, heterogeneous liver function with areas of regional dysfunction or hepatic compensation of local defects can only be assessed non-invasively with imaging techniques.

Abdominal ultrasound is useful for image-based diagnosis of liver function. Ultrasound elastography (US-RTE) can be used to measure liver stiffness, thus allowing an indirect assessment of liver function [7, 8]. However, the diagnostic value of US-RTE is restricted by limited reproducibility and the examiner-dependence of the method [9].

In addition to ultrasound imaging, MRI of the liver currently represents the gold standard of diagnostic methods. Several studies have demonstrated a correlation between hepatic gadolinium ethoxybenzyl-diethylenetriaminepentaacetic acid (Gd-EOB-DTP) uptake and liver function. A common analysis is the measurement of the signal intensity (SI) of T1-weighted volumetric interpolated breathhold examination- (VIBE-) sequences. Regarding SI-based measurements after Gd-EOB-DTPA administration, various SI ratios, such as the relative enhancement of the liver corrected by the spleen or muscle, have been used to assess liver function [10–15]. The evaluation of T1 relaxation time is an alternative approach to the direct measurement of SI and has recently gained attention [12, 16–19]. Haimerl *et al.* recently compared different SI and T1 relaxometry scores to detect the most relevant parameter derived from Gd-EOB-DTPA-enhanced MRI for assessment of liver function [20]. Scores based on T1 relaxometry were superior to SI-based indices for the assessment of liver function.

In addition to T1-weighted sequence analysis, some authors have reported the benefit of diffusion-weighted MRI in analyzing liver function [21–24]. The use of other MRI sequences such as T2-weighted images has not been analyzed.

The purpose of this study was to evaluate the diagnostic performance of multiparametric Gd-EOB-DTPA-enhanced MRI for the estimation of liver classified by the MELD score. Instead of focusing on a single MRI sequence, we examined the full range of available MRI sequences to estimate liver function in a multivariable analysis.

## RESULTS

Patient characteristics stratified by the MELD score are summarized in Table 1. Patients were subdivided into two groups: normal liver function (NLF) and impaired liver function group (ILF). The mean MELD score was 7.7 (± 1.3) for NLF and 14.9 (± 3.7) for the ILF.

**Table 1:** Patient characteristics

	All (*n* = 199)	NLF (*n* = 142)	ILF (*n* = 57)
Age (years)	60.0 ± 12.9	59.8 ± 13.5	60.6 ± 11.3
Sex, *n* (%)			
Male	153 (77)	107 (75)	46 (81)
Female	46 (23)	35 (25)	11 (19)
Weight (kg)	83.1 ± 16.2	84.9 ± 17.7	83.5 ± 12.1
Height (m)	1.7 ± 0.1	1.7 ± 0.1	1.8 ± 0.1
MELD score (range)	9.8 ± 4.0 (6–30)	7.7 8 ± 1.3 (6–10)	14.9 ± 3.7 (11–30)

Table 1 shows the patient characteristics for the subgroups.

Data presented as the means ± standard deviation.

NLF: Normal liver function.

ILF: Impaired liver function.

The logistic regression analysis, with the MELD score as a dependent variable, (Table 2) showed that 6 of the 13 MR sequences including all relative scores were able to classify significantly (*p* < 0.05) liver dysfunction (Table 2).

**Table 2:** Logistic regression

Independent variable	NLF (*n* = 142)	ILF (*n* = 57)	OR (95%-CI)	AUC	*p*-value
T1 mapping 3D					
T1 plain [ms]	770.9 ± 130.1	758.1 ± 143.6	0.99 (0.97, 1.02)^**^	0.584	0.544
**T1 HBP [ms]**	**345.9 ± 93.6**	**460.5 ± 129.2**	1.09 (1.06, 1.13)^**^	**0.751**	**≤0.001**
**RR (plain and HBP)**	**0.5 ± 0.1**	**0.4 ± 0.1**	0.31 (0.22, 0.44)^*^	**0.825**	**≤0.001**
T1 3D VIBE					
**In-phase plain [a.u.]**	**215.5 ± 70.7**	**218.7 ± 42.8**	0.88 (0.81, 0.96)^**^	**0.605**	**0.005**
Opposed-phase plain [a.u.]	237.4 ± 37.6	222.1 ± 37.9	1.02 (0.97, 1.06)^**^	0.574	0.517
fs plain [a.u.]	187.6 ± 32.9	187.5 ± 30.6	1.00 (0.91, 1.10)^**^	0.513	0.987
**fs HBP [a.u.]**	**357.8 ± 82.6**	**283.1** ± **60.5**	0.86 (0.81, 0.91)^**^	**0.772**	**≤0.001**
**RE (fs plain and HBP)**	**0.9 ± 0.3**	**0.5 ± 0.3**	0.67 (0.59, 0.77)^*^	**0.820**	**≤0.001**
**T2 HASTE**	**271.3 ± 70.8**	**240.1 ± 68.2**	0.94 (0.89, 0.98)^**^	**0.622**	**0.006**
T2 BLADE fs	160.9 ± 55.5	168.3 ± 52.1	1.02 (0.97, 1.08)^**^	0.547	0.390
ADC (mm^2^/s)	1.164 × 10^−3^ ± 0.297 × 10^−3^	1.215 × 10^−3^ ± 0.209 × 10^−3^	1.07 (0.96, 1.21)	0.545	0.232

Table 2 shows the results of the logistic regression analyses with the MELD score as a dependent variable.

NLF: Normal liver function; ILF: Impaired liver function.

OR: Odds ratio; CI: Confidence interval; AUC: Area under the curve, *p*: Level of significance.

^*^per 0.1, ^**^per 10 units.

**Figure 1:**
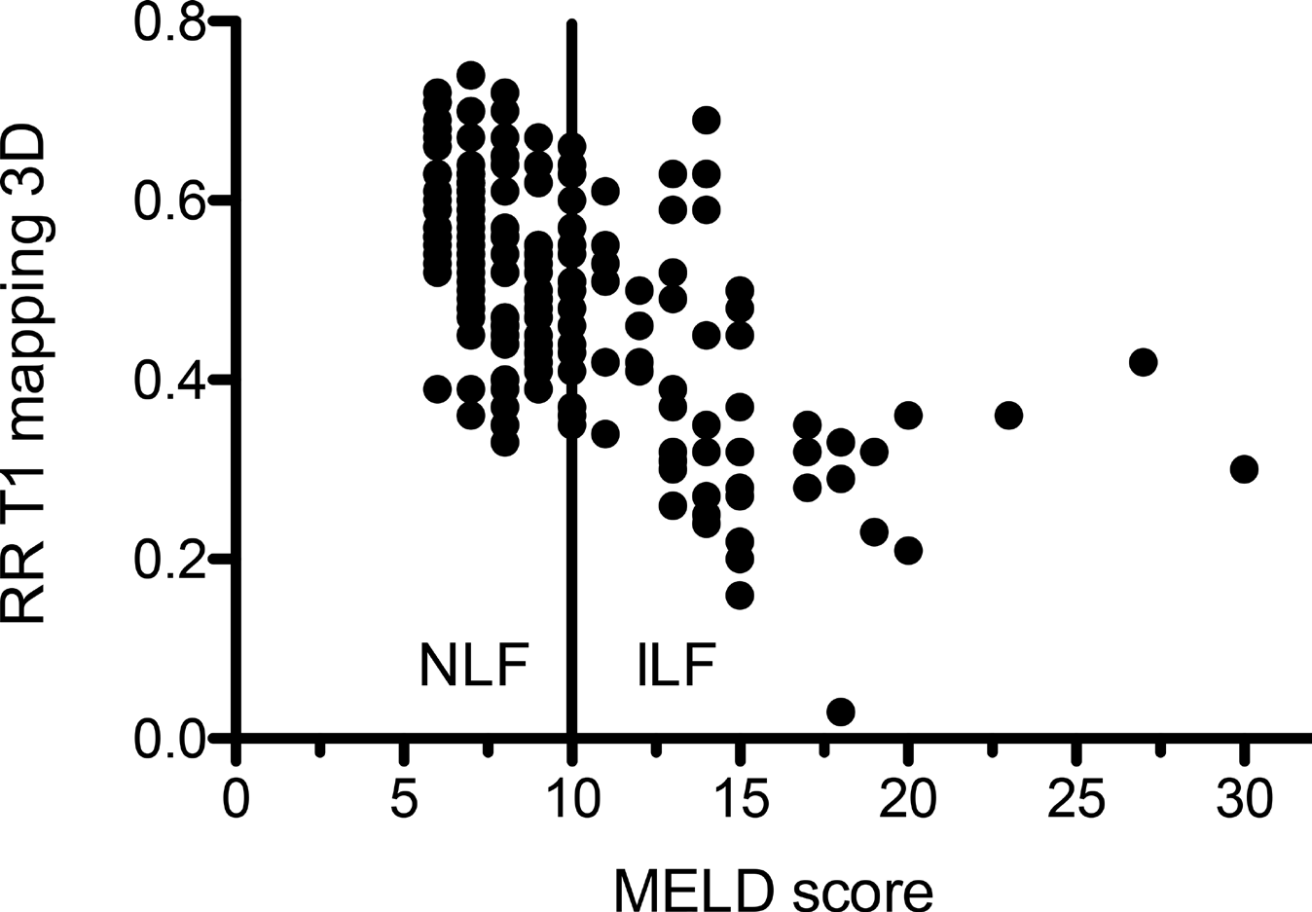
Scatterplot of the reduction rate between plain and contrast enhanced of T1 mapping sequences in correlation to the MELD score The solid line indicates the cut off between normal (NLF) and impaired liver function (ILF).

The MR scores and MR sequences with a significant association were included in a multiple logistic regression analysis. The result is shown in Table 3. In this analysis, only the reduction rate between the 3D T1 mapping sequence remained a significant influencing factor for the MELD score (Figure 1).

**Table 3:** Multiple logistic regression

Independent variable	OR (95%-CI)	*p*-value
T1 mapping 3D HBP	1.03 (0.98, 1.08)^**^	0.307
**RR T1 mapping 3D (plain and HBP)**	0.41 (0.21, 0.82)^*^	**0.012**
T1 3D VIBE in plain	0.92 (0.80, 1.05)^**^	0.219
T1 3D VIBE fs HBP	1.03 (0.93, 1.15)^**^	0.555
RE T1 3D VIBE (fs plain and HBP)	0.92 (0.73, 1.15)^*^	0.446
T2 HASTE	0.96 (0.90, 1.02)^**^	0.214

Table 3 shows the results of the multiple logistic regression analysis with the MELD score as a dependent variable.

OR: Odds ratio; CI: Confidence interval; *p*: Level of significance.

^*^per 0.1, ^**^per 10 units.

## DISCUSSION

Our results showed that liver dysfunction can be assessed by different methods. The logistic regression analysis revealed, that the T1- and T2-weighted images were affected by impaired liver function.

Changes in liver function are often related to liver fibrosis. Liver fibrosis is characterized by destruction of the lobular and vascular architecture and nodular regeneration of liver tissue. Fibrosis of liver tissue results in extracellular accumulation of collagen fibers, proteoglycans, and other macromolecules [25]. Diffusion weighted imaging (DWI) measures the diffusion of water molecules in biological tissues and quantifies the water diffusion processes with the apparent diffusion coefficient (ADC) [26–28]. Theoretically, extracellular collagen fibers, glucosamine, and proteoglycans could inhibit the molecular diffusion of water, resulting in reduced diffusion [26, 28–30]. However, no significant correlation was found in the ADC analysis for this patient cohort.

Notably, the plain in-phase images of the T1-weighted 3D VIBE sequence were able to classify liver dysfunction significantly, while the plain fat suppressed (fs) T1-weighted sequence showed no significant classification. This finding might be due to fat suppression, indicating an impact of fat tissue on liver function. This idea is supported by the fact that the T2-weighted half Fourier single shot turbo spinecho (HASTE) sequence (no fat suppression) also showed a significant result in classifying liver dysfunction. However, in the present study, this influence was not fully defined, and further studies are needed.

Many technical parameters, such as the radiofrequency amplifier, receiver coils, B1-field heterogeneity, repetition times (TR) and respiratory motion, influence absolute values of SI measurements [19, 31, 32].

To overcome this influence, the sequences must be corrected; we calculated the relative change in SI for plain and contrast-enhanced images to measure liver function using T1-weighted images after applying the contrast agent Gd-EOB-DTPA. The liver-specific contrast agent Gadoxetic acid (Gd-EOB-DTPA; Primovist^®^, Bayer Healthcare, Berlin) is an ionic complex consisting of gadolinium (III) and the ligand ethoxybenzyl-diethylenetriaminepentaacetic acid (EOB-DTPA). Gadolinium shortens the spin-lattice relaxation (T1) time in the corresponding tissue, leading to an increase in SI on T1-weighted images [33–36].

The biochemical properties allow a characteristic late phase (HBP) [33–35, 37]. The ethoxybenzyl group promotes the transport of Gd-EOB-DTPA into hepatocytes through organ-anion transporters (OATPB1/B3) located in the sinusoids [38–41], while Gd-EOB-DTPA is excreted at the canalicular membrane by ATP-dependent multidrug resistance protein 2 (MRP2) [42, 43]. Excretion of Gd-EOB-DTPA into the biliary ducts is limited, which causes a temporary enhancement in liver cells [44]. In patients with normal liver parenchyma, the hepatocyte-specific contrast agent shows specific enhancement in the liver parenchyma [37–40]. Since the accumulation of Gd-EOB-DTPA depends on the number of functioning hepatocytes, in the case of liver fibrosis and cirrhosis, the enhancement is reduced, and changes in the liver parenchyma are reflected by Gd-EOB-DTPA uptake [15, 45–48]. While the plain fs T1-weighted images showed no significant value for classifying liver dysfunction, the contrast enhancement in the HBP images showed a significant result. This correlation was even stronger when calculating the relative change in SI. However, neither the fs T1-weighted images during the HBP nor the RE remained significant influencing factors in the multiple logistic regression.

In the plain T1 maps, no significant correlation with liver dysfunction, classified by the MELD score, could be observed.

Controversy currently exists regarding the extent to which the plain T1 relaxation time of the liver is influenced by changes in the liver parenchyma. The T1 relaxation time can be prolonged in plain images in cases of tissue remodeling in liver fibrosis, characterized by inflammation and consequent edema [19, 49, 50]. In contrast, in the advanced stages of liver fibrosis, decreased T1 relaxation times have been reported [51]. This reduction in T1 relaxation time might be due to increased deposition of paramagnetic macromolecules such as collagen tissue that have a lower water content [52, 53].

In simple and multiple logistic regression, we showed that liver dysfunction can be predicted, using the reduction rate in the T1 sequences. Regarding the question of whether SI-based-scores or T1 relaxation time scores should be used, we agree with Haimerl *et al.* [20] - a more reliable outcome can be found using T1 mapping.

In conclusion, Gd-EOB-DTPA-enhanced MRI allowed good prediction of liver dysfunction. It may serve as an appropriate image-based tool for staging liver function before liver surgery, detecting silent disease, or revealing existing disease.

## MATERIALS AND METHODS

### Patient inclusion

The institutional review board approved this retrospective study. Between 03/2016 and 12/2016, 215 Gd-EOB-DTPA-enhanced MRI examinations of the liver were performed. Sixteen patients were excluded from the study due to inability to complete the full MRI protocol or the presence of severe motion artifacts as a result of poor breath-holding technique. Finally, 199 patients were included in this study; the corresponding patient characteristics are listed in Table 1.

### Evaluation of liver function (using established clinical methods)

We used an established clinical scoring system, the MELD score, to assess total liver function. The MELD score is calculated using biochemical blood parameters as follows:

(1) MELD=10*(0.957*ln(serum creatinie)+0.378*ln(total bilirubin)+1.12*ln(prothrombin time, INR)+0.643

To avoid negative scores, any value less than 1 was given a value of 1 (e.g., if the serum bilirubin value was 0.6, a value of 1.0 was used).

Subsequently, the patients were divided into two groups according to their liver function as determined by the MELD score. Following the approach described previously by Verloh *et al.*, a MELD score below ten was considered indicative of normal liver function, and a MELD score above 10 indicated impaired liver function [12]. Patients with impaired liver function (*n* = 57) had different diagnostic assumptions: 26 patients with ethyl-induced liver damage, 17 patients with chronic viral infection, five patients with a non-alcoholic fatty liver disease, three patients with autoimmune disease, six patients with other diseases associated with an impaired liver function such as sclerosing cholangitis.

## MRI

All imaging was performed using a clinical whole body 3T system (MAGNETOM Skyra, Siemens Healthcare). A combination of body and spine array coil elements (18-channel body matrix coil, 24-channel spine matrix coil) was used for signal reception. Images were acquired using various sequences before (native) and 20 min after contrast agent administration (hepatobiliary phase, HBP). All MR sequences with their respective parameters are shown in the Supplementary Materials (Supplementary Table 1).

Gd-EOB-DTPA (Primovist; Bayer Schering Pharma AG, Berlin, Germany) was used as the liver-specific contrast agent and was administered via bolus injection (0.1 ml/kg body weight) with a flow rate of 1 ml/s and was subsequently flushed with 20 ml NaCl.

### Image analysis

The mean SI values on T1-weighted images and the T1 relaxation times on T1 maps of the liver were measured using operator-defined regions of interest (ROIs).

Four circular ROIs were manually positioned by an experienced radiologist in the liver parenchyma at identical locations in all sequences (see the Supplementary Materials for details) at the level of the portal fork, three in right liver lobe, one in the left liver lobe. Visible vessels, liver lesions or imaging artifacts were excluded. The sizes of the ROIs ranged from 1.0 to 2.5 cm^2^, attempting to primarily take the largest diameter. Focal liver parenchyma damage was not found in any of the patients. ROIs were manually adjusted between sequences before and after Gd-EOB-DTPA administration in the case of patient movement. The mean values of these ROIs were then calculated and were considered representative for the entire liver.

Relative changes between the plain and contrast-enhanced series during the HBP were calculated as follows:

(2) $$\text{Relative enhancement of SI}(\text{RE})=\frac{{\text{SI}}_{\text{HBP}}{-\text{SI}}_{\text{plain}}}{{\text{SI}}_{\text{plain}}}$$

(3) $$\text{Reduction rate of the T1 relaxation time}(\text{RR})=\frac{{\text{Tl}}_{\text{plain}}{-\text{Tl}}_{\text{HBP}}}{{\text{Tl}}_{\text{plain}}}$$

### Statistical analysis

The statistical analysis was performed using IBM SPSS Statistics (Version 24, Chicago, IL) and R 3.2.1. All data are presented as means ± standard deviation if not specified otherwise. Logistic regression analyses of MRI sequences were used to determine their assessment of liver function as classified according to the MELD score. Then, multiple logistic regression of all significant values (inclusion criterion: *p* ≤ 0.05) was performed. The statistical significance level was set to 0.05 (two-sided).

## SUPPLEMENTARY MATERIALS TABLE



## Abbreviations

MELD-Score: Model for End-Stage Liver Disease Score
ICG: Indocyanine green
US-RTE: Ultrasound elastography
HBP: hepatobiliary phase
SI: Signal intensity
ROI: Region-of-interest
RE: Relative enhancement
RR: Reduction rate
NLF: Normal liver function
ILF: impaired liver function
VIBE: volumetric interpolated breathhold examination
Gd-EOB-DTPA: gadolinium ethoxybenzyl diethylenetriaminepentaacetic acid
Fs: spectrally fat suppressed
HASTE: Half-Fourier Acquisition Single-shot Turbo spin Echo imaging
