# Supplementary material for: Diagnostic performance of Gd-EOB-DTPA-enhanced MRI for evaluation of liver dysfunction: a multivariable analysis of 3T MRI sequences
Source: Oncotarget. 2018 Nov 20;9(91):36371–8. doi: 10.18632/oncotarget.26368 (PMC6284745; doi:10.18632/oncotarget.26368)
Supplement: Supplementary file 1 [file oncotarget-09-36371-s001.pdf]

Diagnostic performance of Gd-EOB-DTPA-enhanced MRI for evaluation of liver dysfunction: a multivariable analysis of 3T MRI sequences

SUPPLEMENTARY MATERIALS

Supplementary Table 1: All MR sequences with their respective parameters used in this study

Sequence		Acquisition	TR [ms]	TE [ms]	Voxel size	Flip angle	phase
T2 HASTE		Respiratory-triggered	1000	78	measured: 1.5 × 1.3 × 6.0 mm reconstructed: 1.3 × 1.3 × 6.0 mm		plain
VFA T1 mapping 3D*	Dixon-Technique	Breath hold 17 s	5.79	TE 1 = 2.46 TE 2 = 3.69	measured: 3.6 × 2.5 × 4.8 mm reconstructed: 1.3 × 1.3 × 3.0 mm	1°, 7°, 14°	plain + HBP
3D VIBE	Dixon-Technique, In-phase and opposed- phase image	Breath hold 14 s	3.97	TE 1 =1.26 TE 2 = 2.49	measured: 1.7 × 1.3 × 6.0 mm reconstructed: 1.3 × 1.3 × 3.0 mm	9°	plain
3D VIBE	fat suppressed	Breath hold 14 s	3.09	1.17	measured: 1.7 × 1.3 × 4.5 mm reconstructed: 1.3 × 1.3 × 3.0 mm	10°	plain + HBP
T2 BLADE	fat suppressed	Respiratory-triggered	2500	89	measured: 1.3 × 1.3 × 6.0 mm reconstructed: 1.3 × 1.3 × 6.0 mm		After KM
ADC	diffusion weighted, b-Value: 50, 400, 800 (s/mm^2)	Respiratory-triggered	5700	52	measured: 2.5 × 2.1 × 6.0 mm reconstructed: 2.1 × 2.1 × 6.0 mm		After KM

*Prototype technique, the used T1 time has been calculated from the in-phase acquisition.
